# Top quality blastocyst formation rates in relation to progesterone levels on the day of oocyte maturation in GnRH antagonist IVF/ICSI cycles

**DOI:** 10.1371/journal.pone.0176482

**Published:** 2017-05-17

**Authors:** V. S. Vanni, E. Somigliana, M. Reschini, L. Pagliardini, E. Marotta, S. Faulisi, A. Paffoni, P. Vigano’, W. Vegetti, M. Candiani, E. Papaleo

**Affiliations:** 1 Centro Scienze Natalità, IRCCS San Raffaele Scientific Institute, Milan, Italy; 2 Fondazione IRCCS Ca’Granda, Ospedale Maggiore Policlinico, Milan, Italy; 3 Università degli Studi di Milano, Milan, Italy; 4 Division of Genetics and Cell Biology, IRCCS San Raffaele Scientific Institute, Milan, Italy; 5 Università Vita-Salute San Raffaele, Milan, Italy; University of Kansas Medical Center, UNITED STATES

## Abstract

Cycles with progesterone elevation during controlled ovarian stimulation (COS) for IVF/ICSI are commonly managed with a “freeze-all” strategy, due to a well-recognized detrimental effect of high progesterone levels on endometrial receptivity. However, also a detrimental effect of elevated progesterone on day-3 embryo quality has recently been found with regards to top quality embryo formation rate. Because blastocyst culture and cryopreservation are largely adopted, we deemed relevant to determine whether this detrimental effect is also seen on blastocyst quality on day 5–6. This issue was investigated through a large two-center retrospective study including 986 GnRH antagonist IVF/ICSI cycles and using top quality blastocyst formation rate as the main outcome. Results showed that on multivariate analysis sperm motility (p<0.01) and progesterone levels at ovulation triggering (p = 0.01) were the only two variables that significantly predicted top quality blastocyst formation rate after adjusting for relevant factors including female age, BMI, basal AMH and total dose of FSH used for COS. More specifically, progesterone levels at induction showed an inverse relation with top quality blastocyst formation (correlation coefficient B = -1.08, 95% CI -1.9 to -0.02) and ROC curve analysis identified P level >1.49 ng/ml as the best cut-off for identification of patients at risk for the absence of top quality blastocysts (AUC 0.55, p<0.01). Our study is the first to investigate the top quality blastocyst formation rate in relation to progesterone levels in IVF/ICSI cycles, showing that increasing progesterone is associated with lower rates of top quality blastocyst. Hence, the advantages of prolonging COS to maximize the number of collected oocytes might eventually be hindered by a decrease in top quality blastocysts available for transfer, if increasing progesterone levels are observed. This observation extends the results of two recent studies focused on day-3 embryos and deserves further research.

## Introduction

Subtle progesterone elevation throughout Controlled Ovarian Stimulation (COS) for *in vitro* fertilization (IVF) / intracytoplasmic sperm injection (ICSI) is a common occurrence that has gained great attention over the last years, due to a well-documented detrimental impact on endometrial receptivity [1]. In contrast, the majority of the available literature does not suggest any harmful effects of elevated progesterone on oocyte maturation and competence [2–9]. As a consequence, cycles with subtle progesterone elevation are commonly managed with a freeze-all strategy, where the entire cohort of embryos/blastocysts is cryopreserved and a subsequent frozen-thawed transfer is programmed [1,10–11]. However, quality assessment was never the main outcome of recent studies conducted on elevated progesterone. Moreover, the few previous studies focusing on embryo quality were conducted several years ago and most of them included very small numbers of patients [2,12–14]. Thus, major biases and also a lack of statistical power to detect a detrimental effect cannot be excluded. As a matter of fact, this issue has recently become subject of some debate, as Huang et al. have described in a large series of patients a decreased rate of top-quality embryo formation in relation to subtle progesterone elevation, regardless of the age of the woman, the basal FSH, the total dose of gonadotropins used or the duration of ovarian stimulation [15]. This is in accordance with another recent study that found increasing serum P levels (1.60 ng/ml—2.50 ng/ml) to be associated with decreased cumulative live birth rates [16]. Both studies focused on cleavage stage embryos. Evidence on blastocyst quality is conversely very scanty. As blastocyst culture is nowadays largely adopted and quality assessment remains a cornerstone in predicting the outcomes of assisted reproduction [17–18], we deemed of interest determining whether a negative effect of subtle progesterone elevation could be also observed on Day 5–6 of development (blastocyst stage). This issue was investigated with the use of a large two-center retrospective study including 986 IVF/ICSI cycles.

## Materials and methods

### Study design

This was a retrospective, two-centres cohort analysis of patients treated at the infertility unit Centro Scienze Natalità, San Raffaele Scientific Institute, Milan, Italy and at the infertility unit Fondazione Ca’ Granda, Ospedale Maggiore Policlinico, Milan, Italy between January 2013 and August 2016. Inclusion criteria were indication to IVF/ICSI, GnRH antagonist stimulation protocol, cycle with blastocyst culture of the whole cohort of embryos formed and at least one viable blastocyst on day 5–6 and availability of serum progesterone levels on the day of hCG administration. To avoid the impact of intrinsic poor prognosis as a major bias related to blastocyst development, the presence of a poor ovarian response (< 4 oocytes retrieved) [19] and/or severe male factor [Total Motile Sperm Count (TMSC) < 5 million] [20] were both considered as exclusion criteria. All patient gave written informed consent for their anonymized medical records to be used for clinical research purpose and the study was approved by the Institutional Review Board of both participating centres (San Raffaele Scientific Institute, Milan, Italy and Fondazione Ca’ Granda, Ospedale Maggiore Policlinico, Milan, Italy).

### Protocol for ovarian stimulation

All patients were treated with a GnRH antagonist protocol as previously described [21–22]. Briefly, both initial dose of FSH and dose adjustments during treatment were chosen on a case-by-case basis according to patients’ characteristics. A daily dose of 0.25 mg of GnRH antagonist was started on day 6 of stimulation. When three or more leading follicles had reached a diameter ≥17 mm, triggering of ovulation was performed with 10,000 IU of high purified (HP)-hCG or GnRH agonist 0.2 ml in case of risk of OHSS (presence of 25 follicles with a diameter > = 12mm on the day of triggering). Oocyte collection was performed 36 hours after triggering of ovulation. Serum progesterone was systematically assessed on the day of hCG administration in both centers.

### Hormone measurements

At both centres, samples were tested for progesterone level in a Tosoh AIA fluorimetric system with ST-AIA-PACK immunoassay (Tosoh Corporation) as previously described [6]. The assay has a sensitivity of 0.1 ng/ml. Intra-assay and interassay variation coefficients were 11 and 13%, respectively. Besides the internal quality control checks performed daily by the institutional laboratory, the assays were calibrated whenever a new reactive batch was used or whenever an outcome outside the normal range was observed. The assay was used for the entire duration of the study and no corrective intervention had to be performed during the study period.

### Embryo culture and grading

After 2–3 hours incubation in Human Serum Albumin (HSA)-supplemented Fertilization medium (Sage In-Vitro Fertilization, Inc. Trumbull, CT, USA) under oil, selected oocytes were allocated to conventional *in vitro* fertilization or ICSI. For ICSI, denudation of the cumulus oophorus was performed as previously described [23–24]. Inseminated or injected oocyte were grouped cultured in microdrops of equilibrated HSA-supplemented Fertilization or Serum Substitute Supplement (SSS, Irvine, CA, USA)-supplemented Cleavage medium (Sage In-Vitro Fertilization, Inc. Trumbull, CT, USA) under oil respectively. Sixteen-eighteen hours after insemination or ICSI, all oocytes were checked for fertilization as previously described [23] and embryos were cultured until blastocyst stage in SSS (Irvine, CA, USA)-supplemented Blastocyst medium (Sage In-Vitro Fertilization, Inc. Trumbull, CT, USA). As per inclusion criteria, only patients who underwent blastocyst culture of the whole cohort of embryos formed were included, hence no transfer or freezing of day-3 embryos was performed in the context of this study. Blastocyst evaluation was performed according to the Istanbul Consensus [25]. Briefly, on the morning of day 5–6 of development, blastocysts were given a rating based on: (i) the degree of expansion and hatching status (from 1 as early to 4 as hatched blastocyst); (ii) the inner cell mass scored as follows: good- prominent, easily discernible, with many cells that are compacted and tightly adhered together; fair- easily discernible, with many cells that are loosely grouped together; poor—difficult to discern, with few cells; and (iii) the trophectoderm scored as good -many cells forming a cohesive epithelium; fair—few cells forming a loose epithelium and poor—very few cells. According to the Consensus, an optimal embryo at this developmental stage is a fully expanded through to hatched blastocyst with a good inner cell mass and a good trophectoderm. Based on this, ‘top quality’ blastocyst was defined as expanded or hatched blastocyst with both an inner cell mass and multicellular trophectoderm scored good or with only one of the two parameters scored fair and the other one scored good. Blastocysts were never frozen before the expanded stage. In both centers, 95% of the embryo assessment procedures were performed by the supervisors who were unaware of the study hypothesis at the time of evaluation. Although the two units have no personnel in common, they interact strictly for quality control procedures and scientific purposes [23]. In order to set up embryologic studies from the two centers [23,26,27] a shared embryo quality control as well as other subjective assessments (i.e. oocyte quality control, preimplantation diagnosis biopsy control) is undertaken with a biannual frequency. Based on recent literature aimed at quantifying the subjective nature of blastocyst quality assessments [28], Cohen's kappa score [29] was calculated between the two embryologists evaluating blastocyst morphology and was found to be 0.83 (95% CI: 0.60 to 1.00). This value revealed an “almost perfect” inter-observer agreement between the embryologists [30].

### Outcome measure

The main outcome of the study was the top quality blastocyst formation rate per fertilized oocyte (2PN) cultured (%). Fertilization rate (number of 2PN per oocyte used) and blastulation rate (number of blastocysts formed per 2PN cultured) were secondary outcomes.

### Statistics

Data analysis was done with Statistical Package for Social Sciences (SPSS) version 23.0 (SPSS Incl., USA). Univariate and multivariate linear regression analysis of factors related to the top quality blastocyst formation rate were performed and the Beta (B) correlation coefficient and 95% Confidence Intervals (CI) of included variables were calculated. Receiver operating characteristic (ROC) curve analysis was also performed and the optimal cut-off for negative outcome prediction was calculated by maximizing Youden index (J). To avoid potential biases in the results by assuming that any relationship between serum progesterone levels and top quality blastocyst formation rates may be linear, patients were also divided into distinct groups according to the serum progesterone levels on the day of hCG administration (< or ≥ 1 ng/ml, < or ≥ 1.5 ng ml, < or ≥ 2 ng ml, < or ≥ 2.5 ng ml) and non parametric tests (Kruskall-Wallis test for independent samples) were used to compare top quality blastocysts formation rates among the groups. All the tests were two-sided and a p value <0.05 was considered statistically significant.

## Results and discussion

A total of 1415 IVF/ICSI cycles were initially selected. N = 396 cycles were then excluded due to presence of severe male factor infertility (TMSC < 5) and n = 33 cycles were excluded due to poor ovarian response (< 4 oocytes retrieved), leaving n = 986 IVF/ICSI cycles for analysis. Baseline characteristics and outcomes of the included cycles are shown in Table 1.

**Table 1:** Clinical features and IVF/ICSI cycle outcomes.

Clinical feature/outcome	Median (IQR) or number (%)
Age (years)	35.7 (33.1–38.6)
BMI (Kg/m^2^)	21.2 (19.5–23.7)
Duration of infertility (years)	3.3 (2.2–5)
Basal FSH (IU/L)	6.7 (5.5–8)
Basal AMH (ng/mL)	2.6 (1.5–4.7)
Basal AFC	13 (9–19)
TMSC (Million)	49.3 (20.0–100.0)
Sperm volume (mL)	2.5 (2.0–3.5)
Sperm count (M/mL)	39.0 (17.7–55)
Sperm motility (%)	65.0 (50.0–90.0)
Cause of infertility	
Idiopathic	253 (25.6)
Mild/moderate male factor	221 (22.4)
Reduced ovarian reserve	148 (15.0)
Tubal factor	135 (13.7)
Oligo-anovulation	134 (13.6)
Endometriosis	95 (9.6)
Technique used	
IVF	330 (33.5)
ICSI	656 (66.5)
FSH starting dose (IU)	200 (150–225)
FSH total dose (IU)	1800 (1350–2400)
Duration of stimulation (days)	9 (8–10)
Induction of ovulation	
10,000 HP-hCG (IU)	756 (76.4)
0,2 GnRH agonist (ml)	230 (23.3)
E_2_ at induction (pg/mL)	2260 (1574–3098)
P at induction (ng/mL)	0.9 (0.6–1.4)
Retrieved oocytes	10.5 (7–14)
Used oocytes	9 (6–11)
Fertilized oocytes	7 (5–9)
Fertilization rate (%)	80.0 (66.7–91.0)
Blastocysts	3 (2–4)
Blastulation rate (%)	44.4 (28.6–60.0)
Top quality blastocysts (n)	0.6 (0–1)
Top quality blastocysts formation rate (%)	8.5 (0–14.3)

Univariate correlation analysis showed that female age (p = 0.04), sperm motility (p<0.01), P at induction (p<0.01), technique used (ICSI vs IVF) (p<0.01) and fertilization rate (p = 0.01) were significantly related to the top quality blastocyst formation rate (Table 2). All relevant variables retrieved from univariate analysis together with duration of infertility -that showed borderline significance (p = 0.05) on univariate analysis -were subsequently included in the multivariate analysis of factors related to the top quality blastocyst formation rate. Results of the multivariate analysis are also shown in Table 2: after controlling for all the relevant confounders, only sperm motility (p<0.01) and P at induction (p = 0.01) remained significant predictors of top quality blastocyst formation rate.

**Table 2:** Correlation analysis of factors related to the top quality blastocysts formation rate in IVF/ICSI cycles.

Variables	Univariate analysis		Multivariate analysis	
	B Correlation coefficient	*p value*	B Correlation coefficient (95% CI)	*p value*
Female age	-0.22	0.04 *	-0.10 (-0.32 to 0.11)	0.36
BMI	-0.04	0.71		
Duration of infertility	-0.03	0.05 (*)	-0.02 (-0.05 to 0.00)	0.11
Basal AFC	0.11	0.06		
Basal FSH	0.29	0.08		
Basal AMH	0.05	0.70		
Sperm count	-0.01	0.50		
Sperm motility	0.12	< 0.01*	0.11 (0.06 to 0.15)	< 0.01*
FSH total dose	-0.002	0.14		
Induction of ovulation (HP-hCG vs GnRH agonist)	-1.5	0.11		
E_2_ at induction	0.09 x 10^−3^	0.74		
P at induction	-1.4	< 0.01*	-1.08 (-1.9 to -0.02)	0.01*
Retrieved oocytes	-0.002	0.98		
Technique used (ICSI vs IVF)	-3.41	< 0.01*	-1.2 (-3.2 to 0.77)	0.23
Fertilization rate	0.06	0.01*	0.04 (-0.01 to 0.09)	0.16

**Fig 1:**
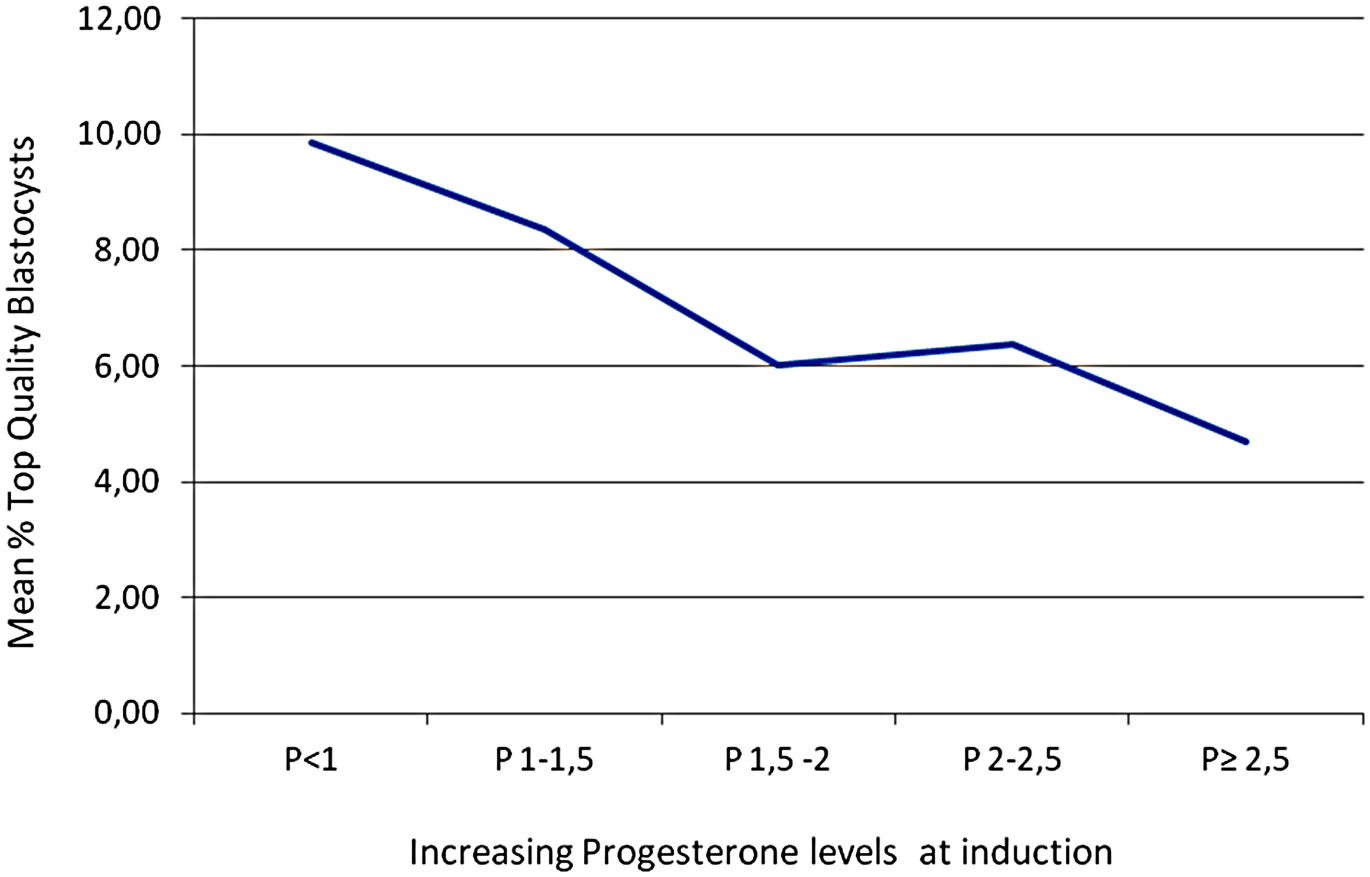
Relationship between serum progesterone levels and top-quality blastocyst formation rate. With an increase in progesterone levels at induction, a decrease in top-quality blastocyst is observed. The decrease in top-quality blastocyst formation rate was found to be statistically significant after dividing patients into groups based on four different cut-off values of serum P levels: < or ≥ 1.0 ng/ml (p <0.01), < or ≥ 1.5 ng/ml (p <0.01), < or ≥ 2.0 ng/ml (p <0.01) and < or ≥ 2.5 ng/ml (p <0.01).

**Fig 2:**
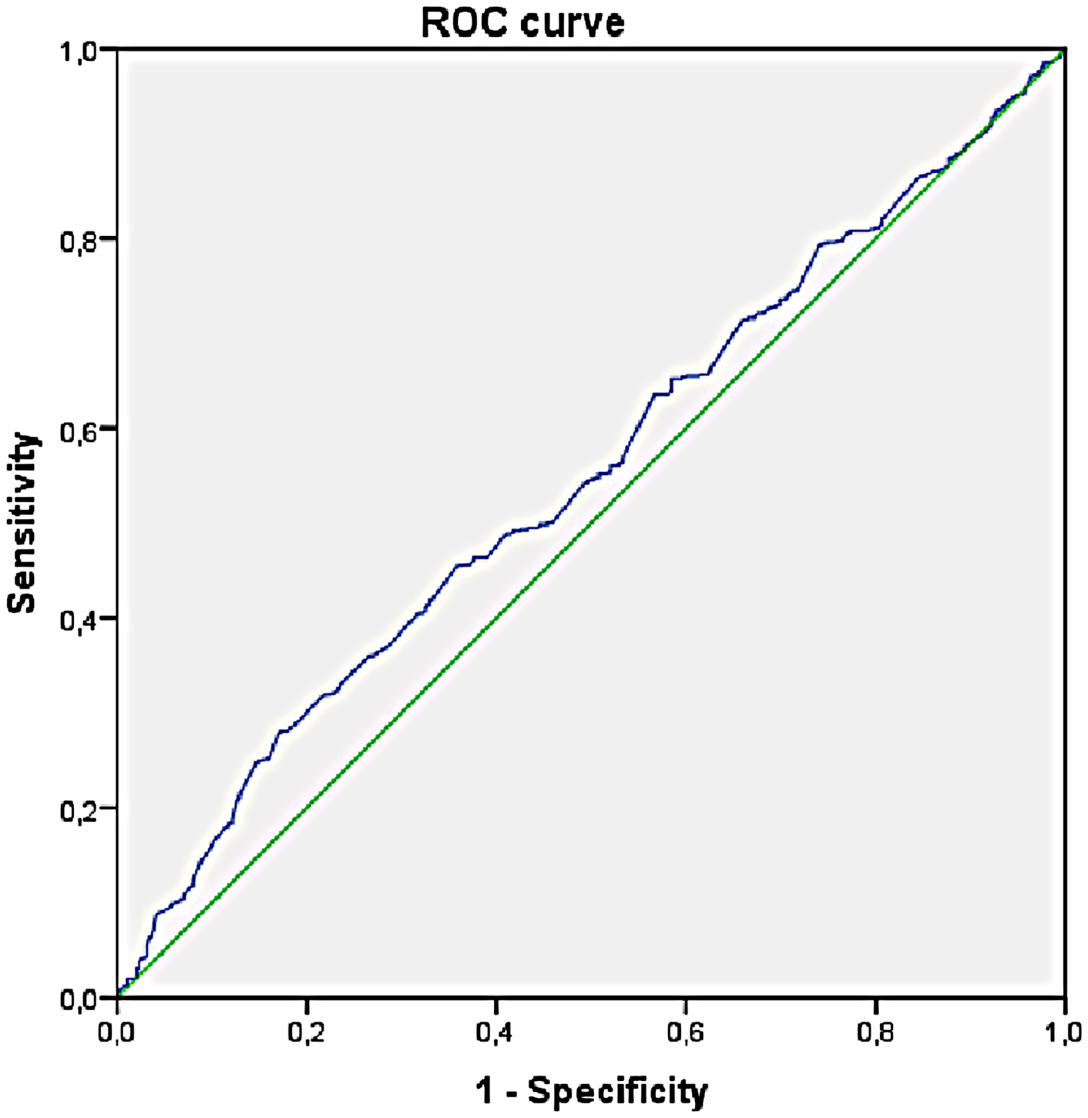
ROC curve analysis for prediction of absence of top-quality blastocyst from progesterone levels at induction. ROC curve analysis for prediction of the absence of top quality blastocyst based on P levels at induction showed a significant AUC = 0.55 (p<0.01). The best cut-off for identification of patients at risk for the absence of top quality blastocyst as obtained by maximization of Youden index is a P level >1.49 ng/ml.

To avoid potential biases in assuming a linear relationship between progesterone levels and top quality blastocyst formation rates, patients were also divided in groups based on increasing progesterone levels (< or ≥ 1 ng/ml, < or ≥ 1.5 ng ml, < or ≥ 2.0 ng ml and < or ≥ 2.5 ng ml) and a significant reduction in top quality blastocyst formation rate in relation to increasing P levels was confirmed with all the four cut-offs used (Fig 1). In contrast, P levels did not correlate significantly neither with fertilization rate (p = 0.17) nor with blastulation rate (p = 0.33) (data not shown). ROC curve analysis for prediction of the absence of top quality blastocyst (i.e. top quality blastocyst formation rate = 0%) based on P levels at induction showed a significant Area Under the Curve (AUC) of 0.55 (p<0.01). By maximizing Youden index (J), we found that a P level >1.49 ng/ml is the best cut-off for identification of patients at risk for the absence of top quality blastocyst (Fig 2).

This large retrospective study demonstrated that elevated serum progesterone on the day of oocyte maturation induction is associated with decreased top quality blastocyst formation rate. This observation is in line with a recent study that found decreased day-3 top quality embryo formation rate in relation to subtle progesterone elevation in IVF cycles [15]. Compared to the work by Huang and colleagues, we deemed relevant to include both IVF and ICSI cycles and we found that the technique used for oocyte fertilization does not significantly influence the detrimental effect of P levels at multivariate analysis. Similarly, we included both cycles where HP-hCG or Gn-RH agonist was used for induction of ovulation, and univariate analysis failed to identify a significant effect of the triggering strategy on the top quality blastocyst formation rate. Taken together, these data suggest that elevated progesterone levels at the end of COS are detrimental to the *in vitro* developmental potential of the oocyte and that no triggering or fertilization technique seems to ‘rescue’ the quality/competence of oocytes exposed to elevated progesterone. There is in general limited data available on the impact of progesterone on embryo quality, but as already discussed by Huang et al., [15], animal studies have documented a progesterone-mediated regulation of oocyte competence [31–34]. Whether these differences in blastocyst quality lead to decreased implantation and pregnancy rates *in vivo* deserves further consideration. As a matter of fact, previous studies have shown that a frozen-thawed embryo transfer can rescue cycles complicated by high P levels at induction, suggesting that the main detrimental effect of P elevation is on endometrial receptivity within the fresh cycle. However, published studies were mostly focused on pregnancy rates in the first frozen-thawed embryo transfer rather than on cumulative pregnancy rates [1,35]. Hence, potential detrimental effects of P elevation on the quality of the whole cohort of blastocysts created has not been thoroughly studied. A recent study that addressed cumulative live birth rates based on P levels indeed found a significant detrimental effect of elevated P on cumulative outcomes, irrespective of poor, intermediate or high ovarian response [16]. Even if another small study recently described similar cumulative live birth rates across groups of patients based on distribution of P levels, results are difficult to interpret because power analysis was not calculated [11]. Interestingly, a recent study has also described that P on the day of trigger is not related to euploidy rates of the cohort of blastocyst created—however only a small number of patients was included and further data on this subject would be of interest [36]. A previous study by our group found similar blastulation rates between groups of patients undergoing a freeze-all strategy with or without elevated progesterone, but was not powered to detect a difference in blastocyst quality [9]. In this larger study, we confirmed that blastulation rates are not impaired by elevated progesterone but we found a significant decrease in top quality blastocyst formation rate in relation to progesterone elevation. The fact that in this analysis we only included GnRH antagonist and not GnRH agonist cycles to some extent also possibly contributes to the differences in the results observed with other recent studies [9,11,16,36]. Our observation may have relevant clinical implications. With the advent of the use of GnRH agonist trigger and freeze all strategies, physicians have become more confident in the presence of a hyper-response and, once decided for a freeze-all policy, they may be tempted to prolong the duration of COS to maximize the number of collected oocytes. This delay may actually increase the rate of subtle elevation of progesterone with hyperstimulation [37]. Based on our results, the advantages of protacting COS might eventually be to some extent hindered by a decrease in top quality blastocysts available for transfer, and a milder stimulation and/or an earlier ovulation triggering might instead be indicated in high responders. More specifically, based on ROC curve analysis in this study, P levels reaching or approximating 1.5 ng/ml could be considered as a warning sign for decreased top quality blastocyst formation rate. In this context, it should be noted that our results apply to GnRH antagonist cycles, that are the currently preferred protocol for maximizing oocyte yeald by prolonging COS and thus might hold relevant clinical implications. However, to determine whether these differences in blastocyst quality lead to decreased blastocyst competence *in vivo* further studies also encompassing cumulative implantation and pregnancy outcomes are required.

## Conclusions

Our study is the first to investigate the top quality blastocyst formation rate in relation to progesterone levels in IVF/ICSI GnRH antagonist cycles, showing that increasing progesterone is associated with lower rates of top quality blastocyst. Our results thus extend the observation of two recent studies focused on Day 3 embryos [15–16] and deserve further research.

## Supporting information

S1 Table Dataset used for the study. (XLSX)
